# Improving Efficiency in Hospital Pharmacy Services: An Integrated Strategy Using the OCTAGON-P Framework and Lean 5S Management Practices

**DOI:** 10.7759/cureus.56965

**Published:** 2024-03-26

**Authors:** Mohammed Sallam, Doaa Allam, Rana Kassem

**Affiliations:** 1 School of Business, International American University, Los Angeles, USA; 2 Department of Pharmacy, Mediclinic Parkview Hospital, Mediclinic Middle East, Dubai, ARE; 3 School of Pharmacy, University of Jordan, Amman, JOR; 4 School of Pharmacy, Queen's University Belfast, Belfast, IRL; 5 School of Business, University of Essex‎, Colchester, GBR

**Keywords:** strategic management, work productivity, innovative strategies, 5s, inventory ‎management, healthcare efficiency, quality improvement study, operation management, hospital pharmacy, lean management

## Abstract

Background

Hospital pharmacy departments have a critical role in the healthcare system, as they aim to provide excellent patient services while also ensuring cost-effectiveness. Lean methodologies are well-known for improving efficiency and quality in various industries, but their impact on healthcare, particularly in hospital pharmacy settings, has not been thoroughly investigated.

Aim

This quality improvement (QI) study aimed to assess the impact of implementing the sort, set in order, shine, standardize, and sustain (5S) methodology using the innovative orientation, coordination, training, awareness, governance, observation, normalization, and promotion (OCTAGON-P) framework on the operations of Mediclinic Parkview Hospital (MPAR) Pharmacy in Dubai, UAE.

Methods

The QI project spanned a period of six weeks, from December 18th^,^ 2023*,* to January 28th^,^ 2024. Throughout this period, a new novel OCTAGON-P framework's eight crucial elements were methodically integrated. Simultaneously, an extensive preparation process encompassing the five stages of the 5S method was carried out.

Results

The findings indicated a notable enhancement in organization, orderliness, cleanliness, medication storage, and workspace standardization. The significant improvement of 217% in terms of organization highlighted the effectiveness of resource arrangement. The orderliness of the workspace saw an increase of 800%, indicating a transformation in the systematic organization. Additionally, cleanliness improved by 138%, demonstrating a significant advancement in maintaining a spotless environment. The standardization of processes experienced a boost of 300%, reflecting a solidified approach to consistent operational methods. These refinements resulted in an overall improvement of 90% from the initial baseline of 20% on the 5S checklist scores. Efficiency gains were observed, with outpatient medication retrieval times reduced by 50%, inpatient times by 40%, emergency prescription serving by 16.7%, and pediatric prescription serving by 11%. The inpatient medication return process saw a 67% improvement. Patient counseling time increased by 23.3%, indicating a more patient-centered approach. Prescription verification and medication expiry checks increased by 50% and 200%, respectively, enhancing the quality of care. Inventory management efficiency improved by 36%, and medication label printing time decreased by 70% with the additional label printers. Installing extra medication label printers was done through the OCTAGON-P framework, specifically in the "orientation" and "coordination" phases. These two initial phases focused on leadership's 5S orientation, management support, and securing additional resources. Therefore, the OCTAGON-P framework provided a structured approach that promoted continuous improvement and sustained lean practices.

Conclusion

This research study presented the remarkable effectiveness of the OCTAGON-P framework in structurally implementing the 5S methodology into hospital pharmacy operations. The findings underscored the potential of lean 5S to enhance and optimize operational efficiency and overall quality within the critical environment of hospital pharmacy settings. Consequently, these improvements can conclusively result in the provision of superior and enhanced patient care, which is truly fundamental and central to the mission and objectives of any healthcare institution.

## Introduction

The sort, set in order, shine, standardize, and sustain (5S) method is straightforward yet impactful for reducing inefficiencies and enhancing productivity within the workplace [1]. The 5S management method, integral to lean principles, has been extensively utilized across diverse industries to streamline workplace organization, enhance efficiency, minimize waste, and optimize quality and productivity by maintaining an organized environment [2]. Waste encompasses any element that does not enhance the process or provide value. The lean philosophy argues that business processes frequently suffer from eight types of waste [3].

Between 1989 and 1991, Takasi Osada pioneered the development of the 5S basis [4]. The 5S methodology originated from five Japanese words that start with “S,” aiming to establish an efficient, safe, and visually managed workspace beneficial to lean operations [5]. The five essential components of 5S are straightforward yet vital to integrate into regular practice.

The 5S framework has been highlighted in the healthcare sector as a viable option for fostering operational enhancements [6]. 5S is used to streamline process flow and improve operational efficiency. The Joint Commission International (JCI) has formulated a series of robust approaches, including lean processes focusing on quality, efficiency, and safety [7], which have assisted UAE hospitals in advancing toward more effective and secure healthcare systems, including medication management and use [8].

According to Huang et al. [1], there is limited data regarding the effects of the 5S management approach on hospital pharmacy operations. Also, until now, the integration of lean 5S principles into healthcare, particularly in pharmacy operations, lacked a distinct, tailored tool.

Jaca et al. [9] suggested creating methods and tools to ease the adoption of 5S principles in organizations across various countries where these practices are less prevalent. Developing programs to assist employees in altering their habits and attitudes towards organization, cleanliness, and workplace enhancement could lead to more practical application of improvement strategies [10]. Therefore, the orientation, coordination, training, awareness, governance, observation, normalization, and promotion (OCTAGON-P) innovative framework was exclusively designed to work in sequence with the 5S scoring system, providing a structured and strategic approach to implementing 5S in hospital pharmacies. Unlike generic applications of 5S, the OCTAGON-P framework is unique in its comprehensive adaptation to the specialized requirements of pharmacy operations, ensuring that each step aligns with the specific goals and challenges of this setting.

The OCTAGON-P framework is an innovative model built with eight essential elements, carefully designed to enhance the implementation of the 5S methodology in a pharmacy setting, targeting the eight pharmacy wastes of lean, including inventory process defects [11] and errors, long service time, inefficient utilization of staff skills, unnecessary movement of medications, unnecessary high medication stock levels, motion waste, unnecessary staff tasks, and unnecessary administrative processes [3].

The process began with "orientation," where establishing a steering group was crucial for guiding and aligning the initiative with the pharmacy's overarching goals. "Coordination" followed, involving the development of a detailed implementation plan to ensure a cohesive and systematic application of 5S. The third element, "training," during weeks 2 and 3, emphasized equipping staff with essential skills and knowledge for effective 5S practice. This was complemented by "awareness," where launching a communication campaign fostered staff awareness and engagement. The fifth element, "governance," involved piloting projects in specific areas of the pharmacy, focusing on medication storage and dispensing, and allowing for the testing and refinement of 5S practices. "Observation" was the sixth step, ensuring the wide-scale adoption of 5S principles through a mass rollout. "Normalization" then drove these methods as standard practices, integral to the pharmacy's daily operations. The final element, "promotion," focused on actively encouraging and sustaining the 5S culture and practices. It involved initiatives to keep the momentum of 5S alive, ensuring that the principles were implemented ad hoc, celebrated, and deeply integrated within the team's workflow. Each element was vital for the successful adoption and implementation of lean 5S, tailored to meet the unique challenges and demands of hospital pharmacy operations [12].

## Materials and methods

Quality improvement project

**Figure 1:**
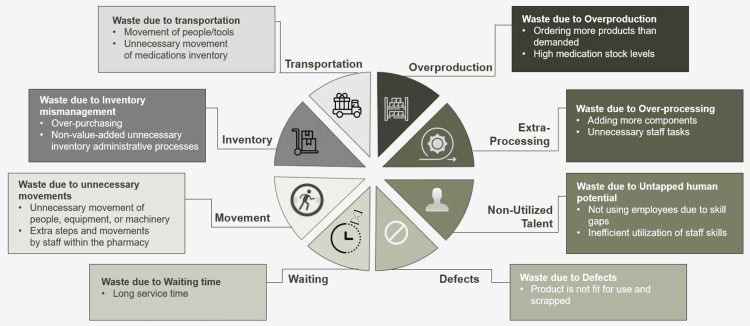
The eight wastes of lean within a hospital pharmacy Image content credit: Dr. Mohammed Sallam

Lean innovation, as a quality improvement (QI) initiative, has substantial positive implications for hospitals [13]. This study documented the transformative effects and benefits of implementing the 5S technique integrated with a new novel OCTAGON-P framework, a quality management tool for organizing hospital pharmacy services. 5S served as a systematic approach aimed at reducing all kinds of waste, optimizing productivity, and enhancing quality by maintaining an orderly workplace and utilizing visual labeling of medication shelves to achieve more consistent operational results [14]. Figure 1 illustrates the eight wastes and inefficiencies commonly encountered in hospital pharmacy operations, per lean management principles.

Project duration, setting, and overview

**Figure 2:**
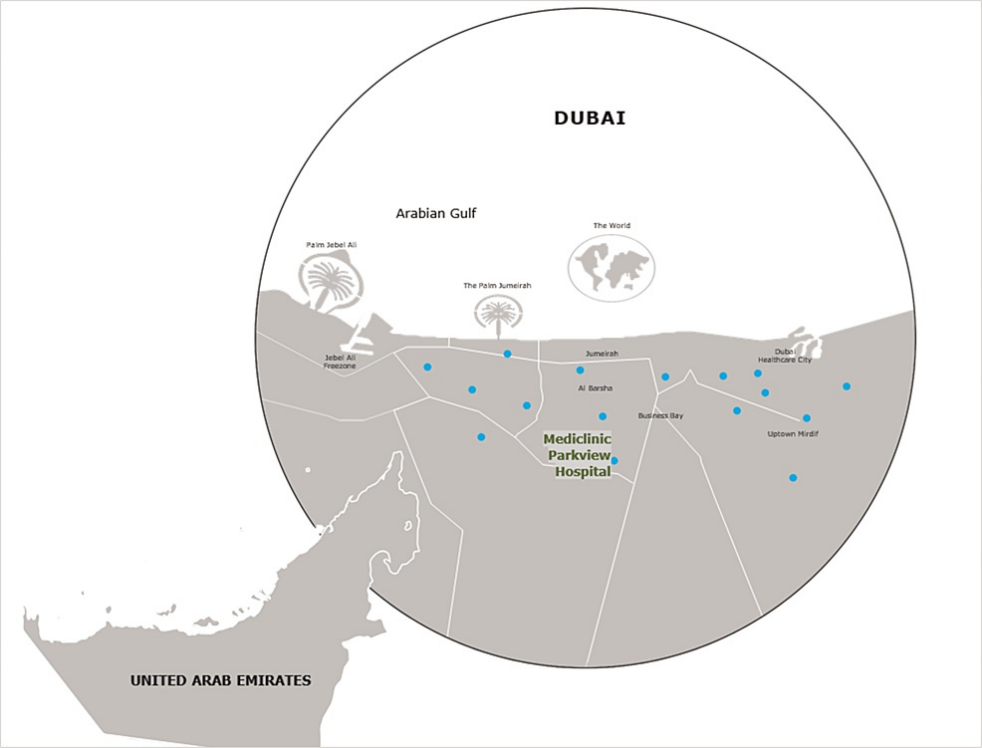
MPAR strategic location in "new Dubai"‎ Image source: Designed by Mediclinic Middle East Marketing Department

The project duration was six weeks, from December 18th, 2023, to January 28th, 2024 (Table 1), and was conducted at Mediclinic Parkview Hospital (MPAR), an 182-bed JCI-accredited tertiary healthcare facility located in the southern side of Dubai, UAE, as shown in Figure 2. MPAR was established in 2018 as Mediclinic Middle East's most extensive greenfield initiative with a broad spectrum of medical services, a range of more than 46 medical specialties, and an extensive in-house 24/7 operating pharmacy that illustrates its commitment to delivering diverse and sophisticated healthcare solutions to the community [15].

**Table 1:** Timeline of the 5S QI project 5S: sort, set in order, shine, standardize, and sustain; QI: quality improvement

Week number	From date	To date
Week 51, 2023	December 18, 2023	December 24, 2023
Week 52, 2023	December 25, 2023	December 31, 2023
Week 01, 2024	January 1, 2024	January 7, 2024
Week 02, 2024	January 8, 2024	January 14, 2024
Week 03, 2024	January 15, 2024	January 21, 2024
Week 04, 2024	January 22, 2024	January 28, 2024

**Figure 3:**
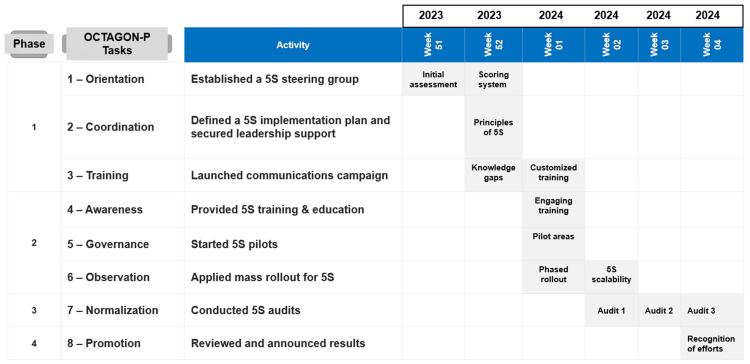
Gantt chart for the QI project OCTAGON-P: orientation, coordination, training, awareness, governance, observation, normalization, and promotion; 5S: sort, set in order, shine, standardize, and sustain Image content credit: Dr. Mohammed Sallam

A Gantt chart served as the study's planning tool, facilitating the visualization and tracking of task progress throughout the project duration [16]. As illustrated in Figure 3, the planning and implementation of the 5S technique, integrated with the OCTAGON-P framework, stretched between December 2023 and January 2024. This period pictured the instrumentation of several critical tasks within the primary structure of the study phases. Activities such as the initial assessment, establishing a steering group, and defining the 5S implementation plan were completed in December 2023. January 2024 was dedicated to further refining the strategy by applying a scoring system for the principles of 5S, thereby ensuring a robust foundation for the continued rollout of this quality management tool in the hospital pharmacy context. The chart captured the transition from planning and groundwork towards tangible actions and audits, reflecting a comprehensive strategy to enhance efficiency, safety, and the quality of service within the hospital pharmacy by utilizing the 5S methodology.

**Figure 4:**
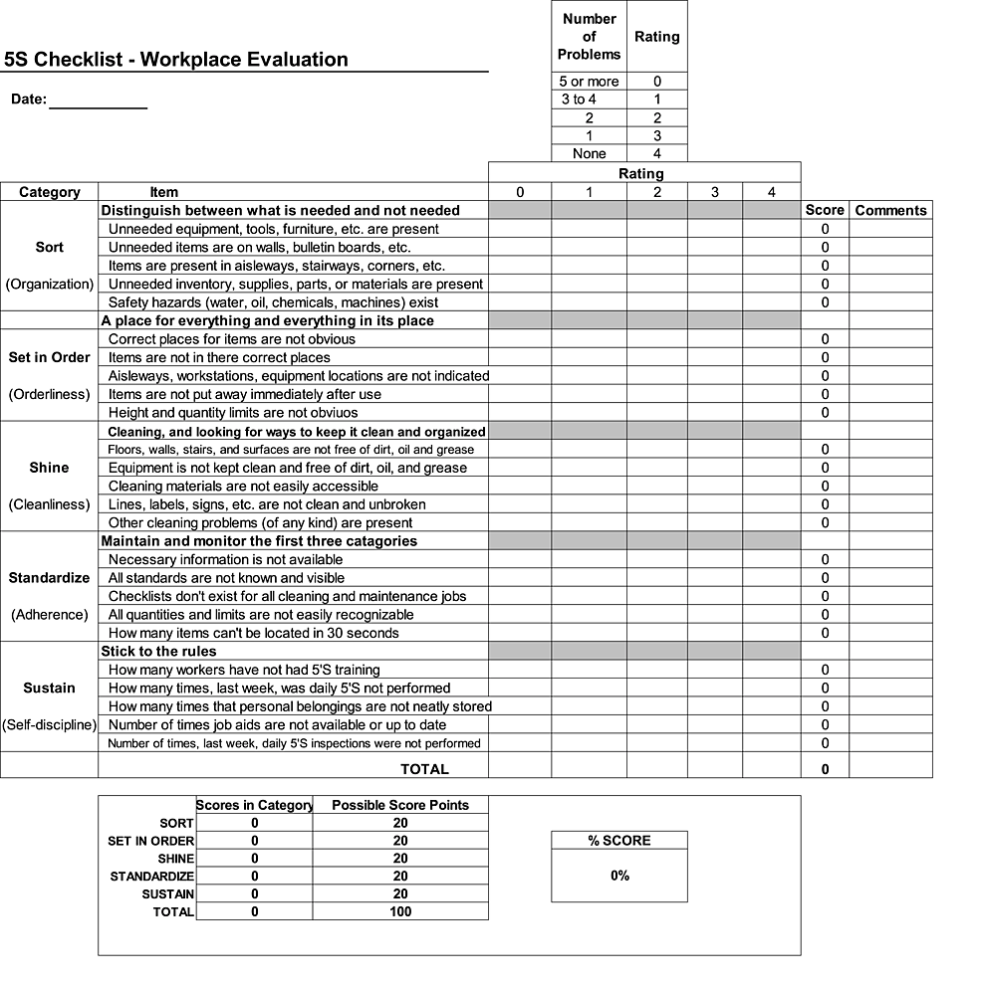
ASQ 5S checklist ASQ: American Society for Quality; 5S: sort, set in order, shine, standardize, and sustain Source: ASQ [5]

The beginning of the implementation phase was characterized by creating a guiding coalition, which involved obtaining the approval of the hospital's top management [17]. The main focus of this phase was to thoroughly educate the hospital administrators about the fundamental principles of the lean methodology, outline the steps involved in its execution, and conduct an initial assessment of the current conditions using a specialized checklist developed by the American Society for Quality (ASQ) (Figure 4) [5]. After this evaluation, the task force proceeded to refine the scoring system. This critical scoring framework was adjusted and adapted from the standards established by Healthcare InfoGuide (Table 2) [18], thus establishing a solid foundation for measuring the anticipated outcomes following the implementation of the pharmacy department. This approach aimed to facilitate a smooth implementation process and align the hospital's strategic direction with the goals of operational efficiency and QI encapsulated by the 5S philosophy.

**Table 2:** 5S scoring guidelines 5S: sort, set in order, shine, standardize, and sustain Source: Adapted from Healthcare InfoGuide [18]

Category	Description	Score	Percentage
Zero effort	No 5S activity	0	0%
Slight effort	Effort from 1-2 people, unorganized	1	20%
Moderate effort	Temporary or superficial attempts at 5S	2	40%
Minimum acceptable level	Team working to standardize improvements	3	60%
Above average results	Excellent level of 5S, room for improvement	3.5	70%
Sustained above-average results	Excellent level sustained over three audits	4	80%
Outstanding results	World-class level of 5S, fully institutionalized	4.5	90%
Sustained outstanding results	World-class level sustained over six audits	5	100%

**Figure 5:**
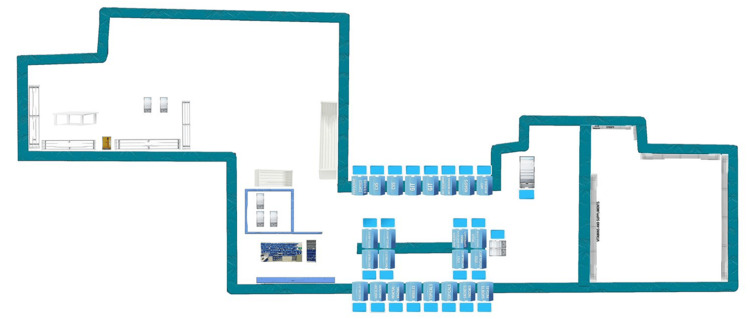
MPAR pharmacy layout MPAR: Mediclinic Parkview Hospital Image credit: Narcotic Pharmacist Muneer Panakada

The second stage defined a detailed 5S implementation plan, which included an assessment of the pharmacy's layout to understand the existing conditions (Figure 5).

This plan was made following comprehensive discussions with all relevant parties, including senior pharmacy personnel and the stock control team. The third stage launched a communications campaign to train the team on the 5S methodology and its potential positive impacts on pharmacy operations and safety, particularly addressing issues like mixed storage in inpatient bins and the non-alphabetical arrangement of medications on outpatient shelves. The fourth step provided education and enrolled the staff, including senior pharmacists and the stock control team. They were briefed on baseline initial assessment information, and the action plan, with their feedback, sought further refinement of the strategy. Moving forward, the fifth stage involved starting the 5S pilot steps to implement the methodology, a process that spanned two weeks (10 working days) and included the medication storage and dispensing areas overseeing the procedure alterations and obtaining input for enhancements. The pilot encompassed all five aspects of 5S: sort (entailed clearing out storage spaces), set in order (restructured the workspace for heightened efficiency), shine (concentrated on upholding cleanliness and equipment upkeep), standardize (established uniform guidelines for tasks), and sustain (aimed to instill a culture of ongoing improvement and adherence to these novel standards). As part of the sixth step, a mass rollout was conducted to expand the 5S practices across the pharmacy. This was followed by the seventh step, which consisted of conducting 5S audits to ensure the effectiveness and adherence to the new systems. The eighth and final step was to continually review and improve the processes, ensuring the sustained success of the 5S implementation in the hospital pharmacy.

**Figure 6:**
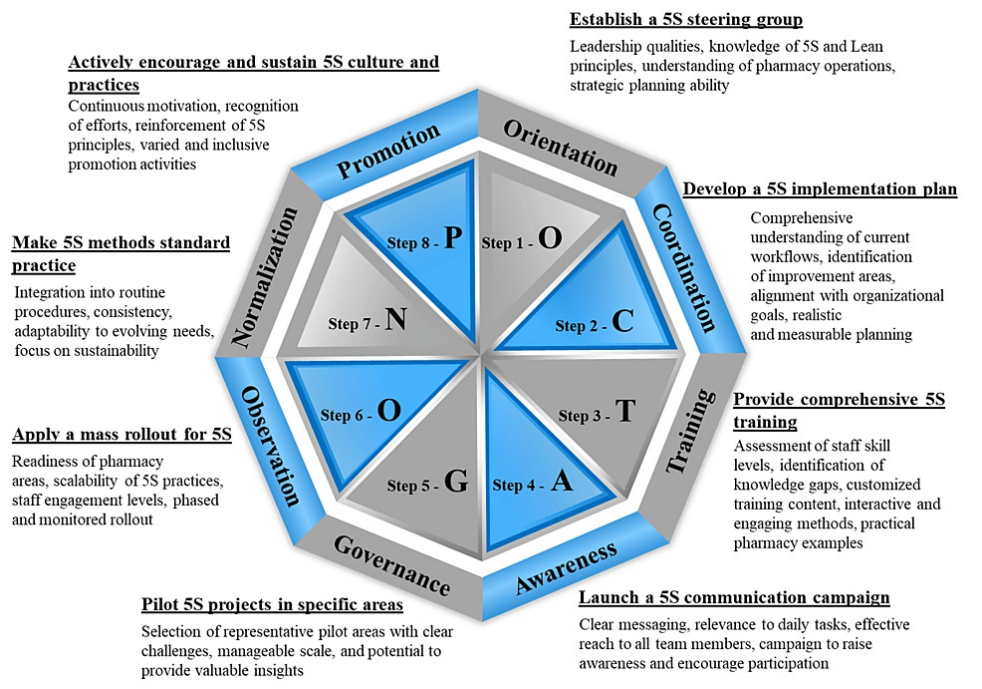
Eight-stage OCTAGON-P framework for pharmacy 5S project OCTAGON-P: orientation, coordination, training, awareness, governance, observation, normalization, and promotion; 5S: sort, set in order, shine, standardize, and sustain Image content credit: Dr. Mohammed Sallam

Figure 6 offers an elaborate visual description of how the 5S methodology was put into practical use within the operational context of the MPAR pharmacy. The implementation of this methodology was guided by the OCTAGON-P framework, which is the author's innovative tool that is structured around eight pivotal elements. Each element strategically focuses on identifying and streamlining inefficiencies commonly encountered in pharmacy operations. The primary objective of this systematic approach was to optimize workflow, eliminate redundant processes, and establish an organizational structure that fosters improved performance and productivity. The framework was specifically designed to address the unique challenges and types of waste that are inherent in pharmacy practice and to create a more adaptable and responsive pharmacy environment.

Ethical considerations

This research did not encompass the involvement of human subjects; consequently, the necessity for ethical approval was exempted. Given the QI evaluative nature of the study design, the Institutional Review Board (IRB) was not required. Additionally, there were no conflicts of interest among the authors, and the project did not receive any commercial funding.

## Results

The quality assessment of 5S integration

**Figure 7:**
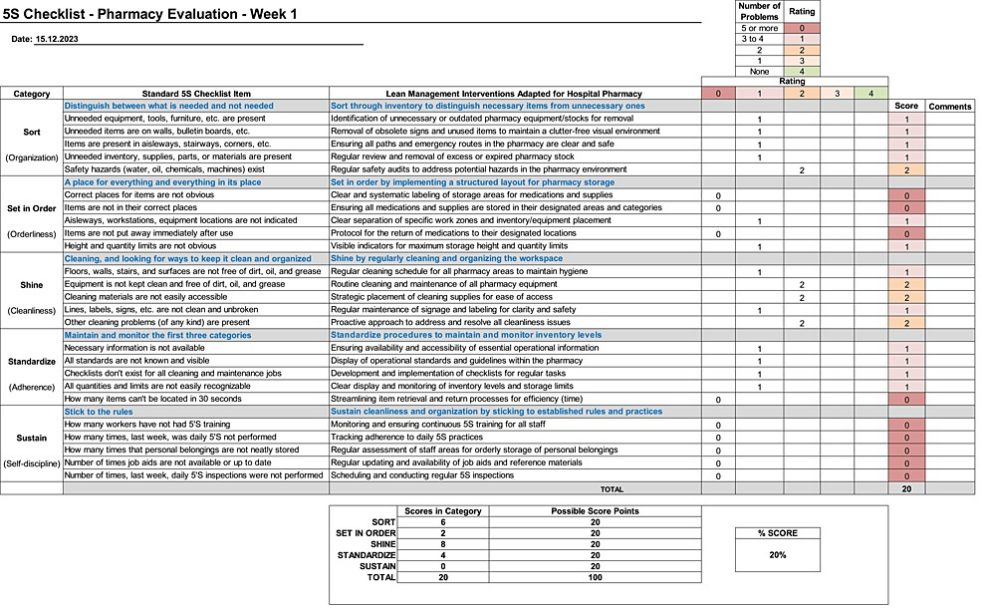
5S initial evaluation checklist for MPAR ‎pharmacy MPAR: Mediclinic Parkview Hospital; 5S: sort, set in order, shine, standardize, and sustain; ASQ: American Society for Quality Source: ASQ [5] Column "Lean management ‎interventions adapted ‎for hospital ‎pharmacy" credit: Dr. Mohammed Sallam

The implementation of the 5S lean methodology within the MPAR hospital pharmacy was systematically structured into five distinct “S” domains. Each domain was designed to create a highly organized, efficient, and safe environment. The process began with an initial assessment of 5S concept integration in the day-to-day operations of a hospital pharmacy using the ASQ checklist tailored for the pharmacy [5]. Figure 7 illustrates that the 5S evaluation checklist commenced with a baseline score reflecting a slight effort, with 20% scoring representing the shy initial engagement in the 5S process within the hospital pharmacy.

The weekly advancements in the 5S initiative at the MPAR pharmacy have demonstrated significant enhancements in both operational efficiency and organizational results.

Sort

The implementation of the first in, first out principle markedly reduced waste due to expired medications, with sorting and discarding unnecessary items streamlining inventory, hence reducing the financial and health consequences that arise from the expiration of medication.

Set in Order

Alphabetical categorization and the use of visual aids like laminated labels for each zone or bin facilitated quicker identification and retrieval of medications, leading to more efficient use of storage space.

Shine

Joint efforts by pharmacy and housekeeping staff significantly enhanced the cleanliness and orderliness of the pharmacy environment, improving both functionality and appearance.

Standardize

The introduction of standardized storage procedures ensured consistent and effective application of the 5S principles, maintaining the organization and efficiency of the pharmacy.

Sustain

A separate and critical aspect of the initiative was the development of a sustainability plan. This included a detailed checklist and regular inspections to ensure ongoing adherence to the 5S methodology, thus maintaining medication efficacy and safety. The sustained efforts helped embed the 5S culture within the pharmacy's daily operations.

**Figure 8:**
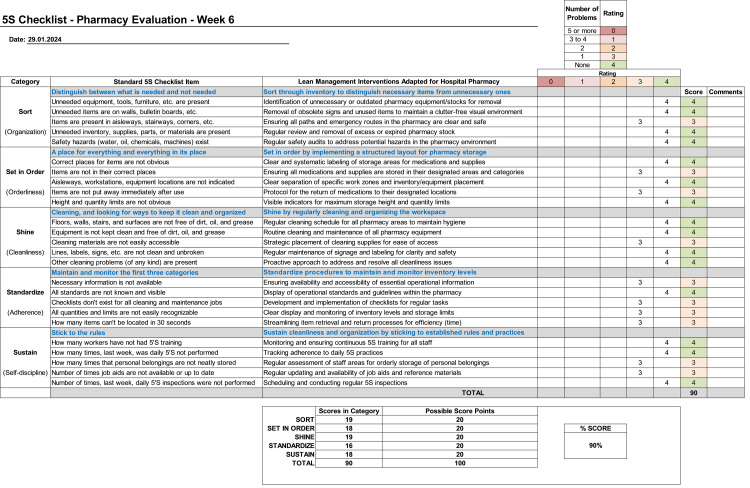
5S third evaluation checklist for MPAR ‎pharmacy MPAR: Mediclinic Parkview Hospital; 5S: sort, set in order, shine, standardize, and sustain; ASQ: American Society for Quality Source: ASQ [5] Column "Lean management ‎interventions adapted ‎for hospital ‎pharmacy" credit: Dr. Mohammed Sallam

The checklist in Figure 8 clearly illustrates the notable improvement in the 5S score of the pharmacy. The score reached an impressive 90% during the third audit, which took place in week 6. This further highlights the exceptional effectiveness of the implemented measures.

Before and after views

**Figure 9:**
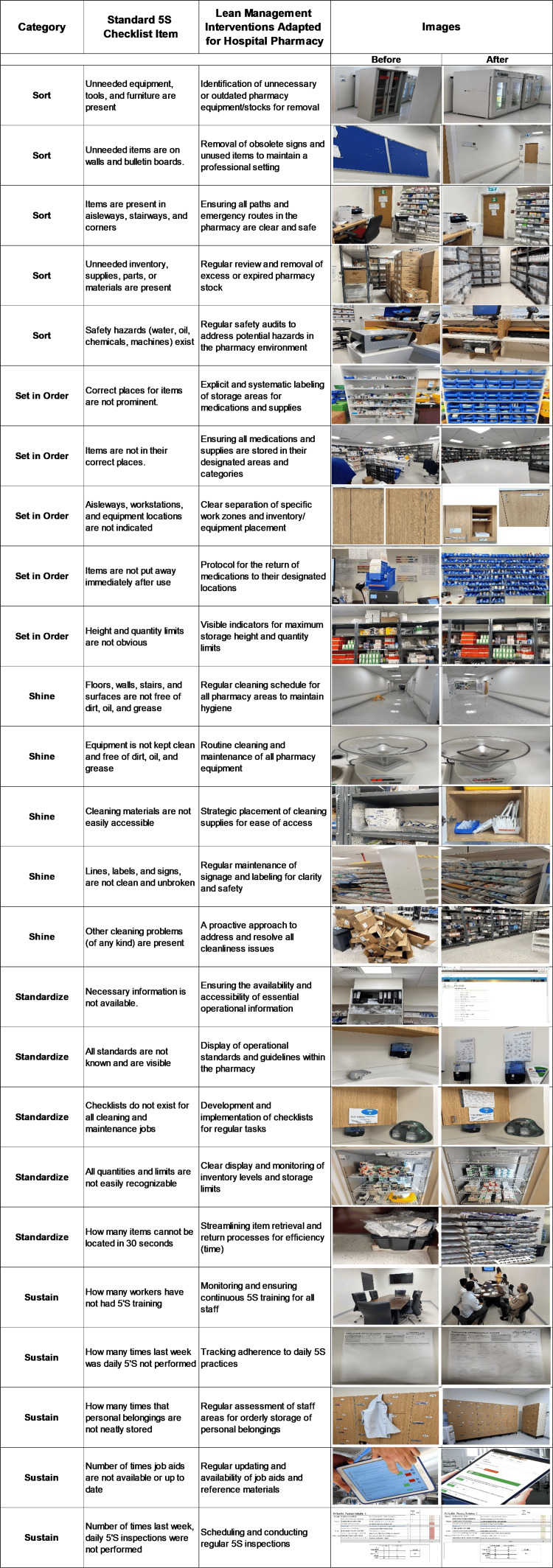
Implementation of the 5S program with before and after views 5S: sort, set in order, shine, standardize, and sustain Image content credit: Dr. Mohammed Sallam

Figure 9 provides a detailed visual representation of the impact of the 5S lean methodology on the hospital pharmacy. The figure uses "before and after" photographs to clearly show the changes brought about by each of the five phases of the 5S process. These images serve as evidence of the transformation the pharmacy underwent. They illustrate the progression from a disorganized state to a well-organized and better functional operation. The pictures highlight the improvement in space utilization, the smoothness of workflows, and the cleanliness and orderliness of the pharmaceutical environment. This visual evidence demonstrates the methodology's effectiveness in creating a productive environment, improving inventory management, promoting safety, and fostering a culture of continuous improvement and attention to detail in healthcare delivery.

Time-based assessment of 5S implementation impact

**Figure 10:**
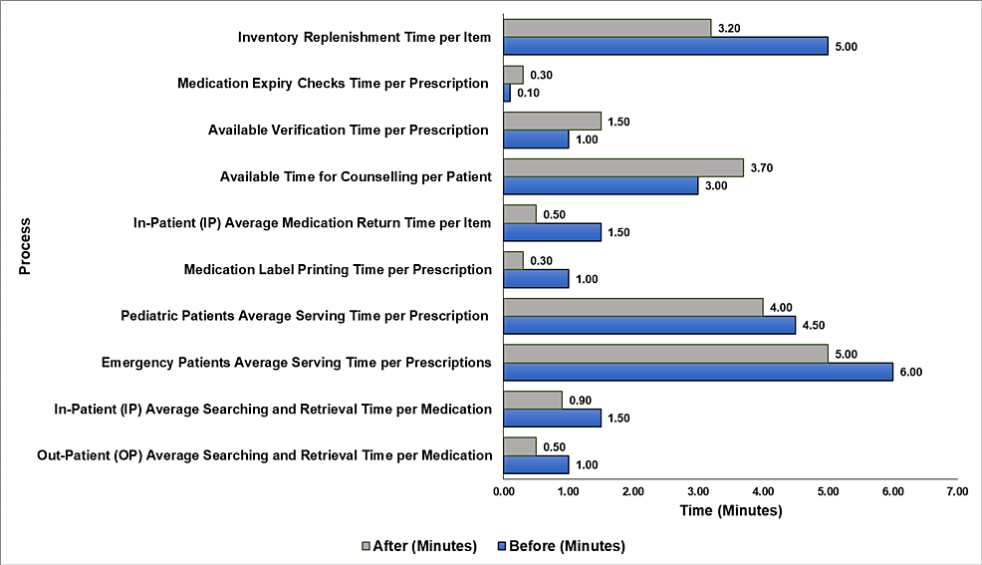
Comparative analysis of time metrics before and after 5S implementation 5S: sort, set in order, shine, standardize, and sustain

Implementing the 5S methodology within the hospital pharmacy setting yielded notable efficiency improvements that are both quantifiable and indicative of enhanced operational performance. The data revealed that medication retrieval times for outpatients were halved, resulting in a 50% improvement, while inpatient retrieval times saw a 40% reduction. Emergency prescription servicing was expedited by 16.7%, with pediatric prescriptions being served 11% more quickly. The process for returning medications for inpatients was optimized, leading to a remarkable 67% decrease in time. The extended duration for patient counseling, which increased by 23.3%, suggests a more patient-centric service approach. Simultaneously, a meticulous emphasis on prescription quality and safety was observed, with verification times increasing by 50% and medication expiry checks by 200%, ensuring a higher standard of care. Inventory management processes also benefited from the 5S implementation, with a 36% reduction in replenishment time, signifying a leaner, more efficient operation. Finally, the introduction of four additional label printers in the pharmacy significantly impacted the efficiency of the medication label printing process. This enhancement resulted in a remarkable 70% reduction in the average printing time per prescription, decreasing from 1 minute to 0.30 minutes. Figure 10 presents a comparative analysis of time metrics before and after the implementation of the 5S methodology.

## Discussion

While the 5S lean methodology originated and gained a reputation within Toyota's automobile manufacturing, its application has now seen widespread adoption across various industries globally. It acts as a fundamental aid to other operational improvements like just-in-time production, total quality management, and six-sigma initiatives [19]. Furthermore, its implementation is crucial in enhancing the workplace, making it a more pleasant and productive environment for employees [20].

In non-lean systems, unnecessary movement of people and equipment during work processes leads to increased fatigue, lower productivity, and a higher incidence of quality errors [21]. This study illustrated the effective implementation of the 5S methodology in enhancing the management, accessibility, and safety of hospital pharmacy and medication storage [22].

Incorporating the OCTAGON-P framework offered a structured, strategic approach specifically tailored to the unique environment of pharmacy operations. It guided the process through critical stages, starting with orientation for setting the initiative's direction, followed by coordination for detailed planning. Training and awareness phases ensured that staff were skilled and engaged, while governance and observation facilitated the testing and broad adoption of 5S practices. Normalization and promotion's final stages embedded these practices into daily operations and nurtured a sustained 5S culture. The OCTAGON-P framework's role was instrumental in the smooth and effective integration of 5S principles, contributing significantly to the observed improvements in process efficiency and operational excellence.

Singh et al. [23] and Gupta et al. [24] observed that implementing 5S contributed to fostering a positive mindset among staff regarding their duties and encouraged others to adopt the 5S methodology.

The 5S methodology's focus on waste elimination has resulted in clear process enhancements within the hospital pharmacy [22]. By identifying and addressing inefficiencies such as time-consuming searches for medications and bulky access and return of items, the pharmacy experienced a notable streamlining of operations. Introducing a practical storage solution characterized by alphabetically arranged medications and visually organized spaces was instrumental in this transformation. This strategic reorganization significantly reduced the travel distance for medication allocation and the time required to fill prescriptions, thereby decreasing both serving and waiting times.

A detailed time analysis post-implementation of the MPAR 5S program underscored these operational improvements. Reduced medication retrieval and serving times, quicker medication return processes, and increased time allocated for patient consultations all indicate a more efficient and patient-focused pharmacy environment. This resource augmentation streamlined the label production process by alleviating bottlenecks in the printing queue, thereby speeding up prescription preparation and contributing to a notable decrease in overall patient waiting times. The modest increase in time spent on prescription verification and medication expiry checks suggests a deliberate shift towards a higher patient care and medication safety standard.

Table 3 demonstrates the application of the OCTAGON-P framework in targeting the eight wastes of lean within a hospital pharmacy setting, aligning strategies for identifying and eliminating waste, and highlighting the results achieved.

**Table 3:** Lean waste reduction in hospital pharmacy: strategies and results OCTAGON-P: orientation, coordination, training, awareness, governance, observation, normalization, and promotion

The Wastes of Lean in Hospital Pharmacy	Type	Description of Waste	Lean Operational Process Improvement Action Applied	OCTAGON-P Framework Step Applied	Results
Process defects	Defects	Prolonged retrieval times with a high chance of picking errors for outpatient and inpatient medications due to suboptimal organization and medication arrangements	Implemented error-preventing techniques and optimized storage layout and labeling for easy access and visibility	Orientation and coordination	Achieved a 50% reduction in outpatient and a 40% reduction in inpatient medication retrieval times
Long service time	Time	Extended duration in serving patient prescriptions	Streamlined the prescription filling process and implemented efficient queue management systems	Training and observation	Decreased average serving time by 16.7% for emergency prescriptions and by 11% for pediatric prescriptions
Inefficient utilization of staff skills	Talent	Underuse of pharmacist and staff expertise	Optimized task allocation based on skills and expertise and provided continuous training and development	Training	Time saved on unnecessary tasks was reallocated to patient counseling
Unnecessary ‎‎movement of medications	Transportation	Excessive physical movement for medication retrieval	Redesigned pharmacy storage areas to minimize movement and ensure frequently used items were easily accessible	Coordination and Governance	Reduced outpatient and inpatient medication retrieval times by 50% and 40%
High medication stock levels	Inventory	Overstocking leads to increased storage costs and expired drugs	Implemented material requirements planning inventory system and conducted regular inventory audits to reduce excess stock	Governance and normalization	36% improvement in inventory replenishment efficiency
Extra steps and ‎movements by ‎staff within the ‎pharmacy	Motion	Extra steps and movements by staff within the pharmacy	Conducted workflow analysis and rearranged activities to minimize unnecessary movement	Awareness and normalization	Reduced inpatient medication return time by 67%
Unnecessary staff tasks	Efficiency	Redundant tasks that do not add value	Identified and eliminated non-value-adding activities, streamlined workflows, and reduced repetitive tasks	Observation and promotion	Time saved on unnecessary tasks was reallocated to patient counseling
Unnecessary inventory administrative processes	Process	Extra processing steps in administrative tasks	Simplified administrative processes	Training and promotion	Streamlined administrative tasks, increasing verification time for quality

The above improvement illustrated the synergistic effect of resource optimization combined with the 5S methodology in enhancing pharmacy operations. These process enhancements align with the broader objectives of quality assurance in healthcare. The application of the 5S methodology was shown to contribute to cost-effective outcomes by optimizing inventory management and maximizing space utilization. Moreover, adopting safety storage measures emerged as a preventative approach to error reduction, further underscoring the value of the 5S framework in fostering a more efficient, safe, and patient-oriented pharmacy service.

The study addressed several operational inefficiencies in the hospital pharmacy by applying lean operational process improvements in conjunction with the new novel OCTAGON-P framework, implementing proactive error-preventing techniques, and optimizing storage layout and labeling for process defects for better accessibility and visibility. The prescription filling process was streamlined to reduce long service times. To address the inefficient utilization of staff skills, we optimized task allocation based on skills and expertise and provided continuous training and development. To minimize unnecessary medication movement, the pharmacy storage areas were redesigned to reduce physical movement and ensure easy access to frequently used items. High medication stock levels were addressed by implementing a material requirements planning inventory system and conducting regular inventory audits to reduce excess stock. Lastly, to tackle extra steps and movements by staff within the pharmacy, we conducted a workflow analysis and rearrangements to minimize unnecessary movement and identify and eliminate non-value-adding activities, streamlining workflows. Each process improvement was vital to enhancing the efficiency and effectiveness of the pharmacy's operations.

Study limitations

Although the 5S system may suit numerous businesses across various industries, it is not a universally applicable solution. This quality improvement project focused on findings specific to MPAR in Dubai, UAE, and did not capture other hospital pharmacy settings. Additionally, the study's short-term evaluation period of six weeks may not fully capture the long-term sustainability and effectiveness of the implemented changes [20]. Moreover, potential biases in observational data collection via the checklist could influence the interpretation of results. Factors such as resistance to change, staff project engagement, technology, or policies may also impact project outcomes [25]. Addressing these limitations is crucial for a comprehensive understanding of the project's outcomes and implications for future initiatives in hospital pharmacy operations.

Future research

This study offered valuable visual evidence regarding the benefits of implementing the 5S methodology in hospital pharmacy settings, illustrating enhancements in workspace organization, cleanliness, and standardization. These findings underscored the effectiveness of lean management practices in improving operational efficiency and the quality of services within healthcare facilities. It is crucial for future research to explore the potential impact on patient safety. Future studies could build on our findings by directly examining how methodologies like 5S and the OCTAGON-P framework influence patient safety outcomes in hospital pharmacy settings, like medication error rates, adverse drug events, or patient therapy outcomes. Additionally, there is a clear requirement for future empirical investigations to explore and clarify the execution of innovative management systems and technological applications driven by artificial intelligence [26]. These endeavors seek to enhance the practical value of lean systems and assess their real impact on decision-making in quality improvement initiatives and overall company performance in the healthcare industry, with a specific focus on the pharmacy sector. Such research would provide valuable insights for healthcare organizations seeking to optimize operations and enhance patient outcomes.

## Conclusions

The successful implementation of the 5S methodology in MPAR pharmacy within a comparatively brief timeframe is a testament to the efficacy of this lean universal tool alongside the novel MPAR OCTAGON-P framework. This unique model is designed for adaptability and can be seamlessly integrated into diverse pharmacy environments, regardless of location. With its structured approach, the OCTAGON-P framework guided healthcare professionals through each stage of 5S implementation, from the initial orientation phase, including leadership support, to promoting sustained quality improvement practices. This approach maximized operational effectiveness and promoted an ongoing advancement culture, prioritizing patient safety and care quality. It offers policymakers and facility managers a strategic tool to improve healthcare delivery substantially, ensuring that quality and efficiency are at the forefront of health policy. The novel OCTAGON-P framework stands out as an inspiration for healthcare and pharmacy institutions aiming to achieve excellence in service and care.

## Figures and Tables

**Figure 11:**
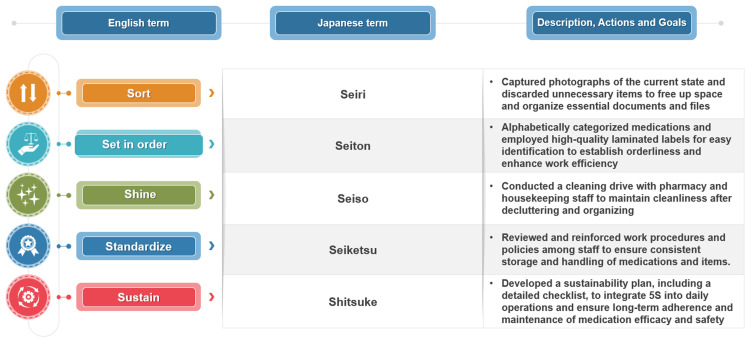
‎5S implementation stages at MPAR pharmacy Image content credit: Dr. Mohammed Sallam

**Figure 12:**
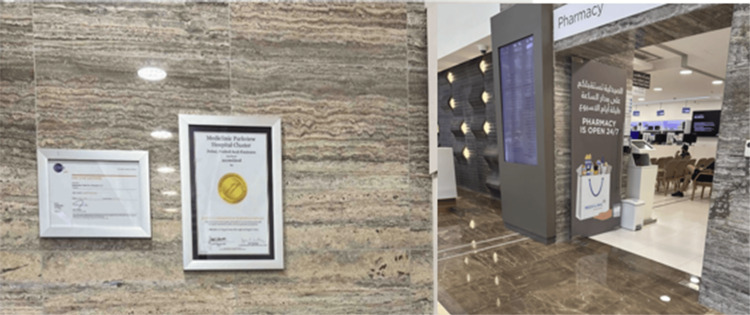
Enhancing both the aesthetic appeal and the functional workspace Image credit: Dr. Mohammed Sallam

**Figure 13:**
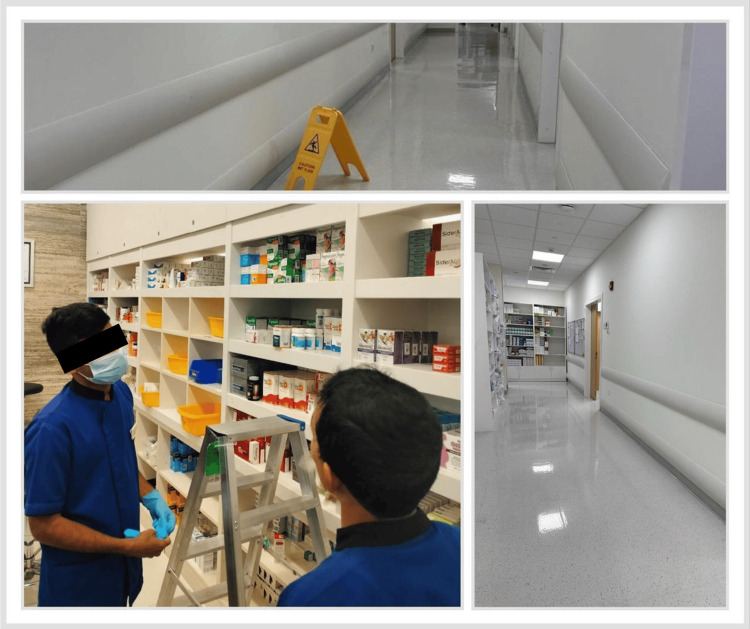
Pharmacy deep cleaning initiative Image credit: Dr. Mohammed Sallam

**Figure 14:**
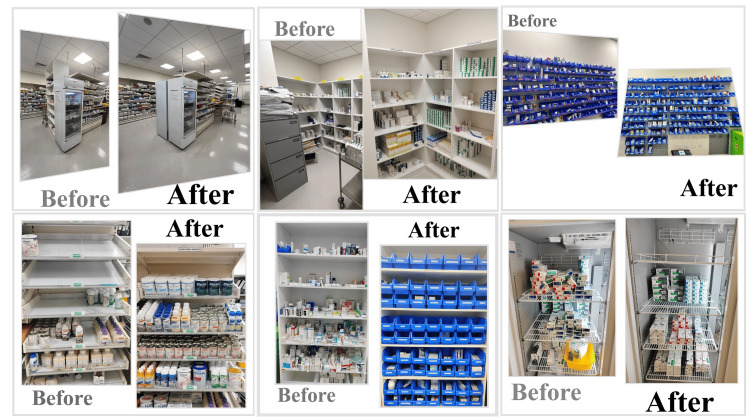
MPAR pharmacy before and after views MPAR: Mediclinic Parkview Hospital Images credit: Dr. Mohammed Sallam

## Appendices

Additional visuals

A set of comparative images showcases the implementation of lean 5S in the pharmacy department of MPAR, showing both the pre-implementation stage and the subsequent post-implementation stage actions and results. Figure 11 summarizes each stage's key actions and goals in the 5S initiative for the hospital pharmacy.

Figure 12 shows enhancements in both the aesthetic appeal and the functional workplace.

Figure 13 indicates the collaborative effort to promote cleanliness, involving both the pharmacy employees and housekeeping personnel.

 Figure 14 shows more before and after pharmacy images.
